# The Detrimental Impact of Bisphenol S (BPS) on Trophoblastic Cells and the Ishikawa Cell Lines: An In Vitro Model of Cytotoxic Effect and Molecular Interactions

**DOI:** 10.3390/biomedicines13081938

**Published:** 2025-08-08

**Authors:** Eirini Drakaki, Despoina Mavrogianni, Anastasios Potiris, Stavroula Xydi-Chrysafi, Panagiotis Kotrotsos, Nikolaos Thomakos, Alexandros Rodolakis, Georgios Daskalakis, Ekaterini Domali

**Affiliations:** 1 First Department of Obstetrics and Gynecology, Alexandra Hospital, Medical School, National and Kapodistrian University of Athens, 115 28 Athens, Greece; eirinidrak@med.uoa.gr (E.D.); dmavrogianni@med.uoa.gr (D.M.); staxydchrys@med.uoa.gr (S.X.-C.); panosktr@med.uoa.gr (P.K.); nthomakos@med.uoa.gr (N.T.); arodolak@med.uoa.gr (A.R.); gdaskalakis@med.uoa.gr (G.D.); kdomali@med.uoa.gr (E.D.); 2 Third Department of Obstetrics and Gynecology, University General Hospital “ATTIKON”, Medical School, National and Kapodistrian University of Athens, 124 62 Athens, Greece

**Keywords:** endocrine-disrupting chemicals (EDC), bisphenol S (BPS), trophoblast cells, Ishikawa cell line, DNA methyltransferase (DNMT), NANOG silencing

## Abstract

**Background/Objectives**: Bisphenols (BPs) and especially bisphenol S (BPS), an analog of bisphenol A (BPA), are widely used and induce oxidative stress, resulting in the inhibition of cell proliferation and induction of apoptosis which all are crucial for reproduction, the progression of pregnancy, and fertility. The present study integrates trophoblastic cells as an in vitro model to provide evidence and investigate the molecular interactions regarding placenta-related pregnancy complications after cytotoxic exposure to BPS. **Methods**: Human endometrial epithelial adenocarcinoma Ishikawa cell lines and trophoblastic cells were cultured. Cells obtained from the cultures were divided into plates and incubated for 24 h with different concentrations of bisphenol S (BPS). Cell viability was measured using the Countess Automated Cell Counter and the viability of Ishikawa cells was assessed after 48 h and for trophoblasts after 24 h. The effect of siRNA on *NANOG* expression was evaluated using qRT-PCR. Quantification of DNMT and NANOG was performed by qPCR and the G6PD gene was used as an internal control. **Results**: Real-time PCR results showed that the expression of the *DNMT1* gene varies depending on the concentration of BPS in trophoblastic cells. In Ishikawa cell lines, real-time PCR results showed that *DNMT1* gene expression was higher due to cell increase, but the measured fold change did not differ significantly. Data analysis indicated a statistically significant difference between CpDNMT1 in trophoblasts with and without BPS, where higher values were observed in the case of BPS presence (*p* = 0.019). The largest difference was observed between CpDNMT1 trophoblasts without BPS and CpDNMT1 Ishikawa with BPS (*p* < 0.001). Silencing the *NANOG* gene resulted in a reduced expression of *DNMT1*, while the *G6PD* gene was still detected. **Conclusions**: The results of this study highlight the cytotoxic effects of BPS and consequently its effect on trophoblast viability. The results of NANOG-DNMT1 gene expression related to BPS exposure reinforces our understanding of EDC-induced placental dysfunction.

## 1. Introduction

Exposure to a variety of harmful chemicals occurs daily through their diffuse in water, air, and everyday products and, according to epidemiological studies, seems to be unavoidable [1,2,3]. This exposure can cause immediate or delayed disruption to physiological functions which can last for a lifetime [1]. Although there is a battery of substances that can interact with the endocrine system without disrupting it, endocrine-disrupting chemicals (EDC) are synthetic and natural compounds that can mimic or antagonize endogenous hormone responses and may have detrimental effects [4]. EDCs cause tissue-specific estrogenic effects disrupting estrogen receptor α-dependent transcriptional signaling pathways and influencing the modulation of growth factors [5].

Bisphenols (BPs) are widely used in the production of polycarbonate plastics and epoxy resins. Bisphenol S (BPS), an analog of bisphenol A (BPA), is a phenolic ring compound [6]. Exposure to BPS is several times higher than the tolerable daily intake in many countries [7]. BPS induces oxidative stress, resulting in inhibition of cell proliferation and induction of apoptosis which all are crucial for reproduction, the progression of pregnancy, and fertility per se [6,8,9,10]. Moreover, bisphenol exposure is detectable in maternal blood, urine, and the placenta [11,12,13]. Bisphenols have the potential to alter placenta cell functions, like proliferation, differentiation, invasion, and hormone synthesis, leading to pregnancy complications such as preeclampsia, preterm birth, miscarriage, and fetal growth restriction [14,15,16,17,18,19,20,21].

These circulating compounds in the maternal bloodstream can not only be transferred across the placenta but also modify the placental function itself. The placenta as part of the endocrine system and also an organ produces a range of hormones and cytokine factors that act on maternal and fetal tissues [22]. Although the fetal placental cells, trophoblasts (TB), have the ability to detoxify many chemicals, they remain vulnerable to a myriad of others [23]. According to previous studies, the susceptibility of cultured cell types to EDC-induced epimutagenesis has been shown. However, these studies were focused on cancer cell lines characterized by altered cellular signaling and not necessarily corresponding to model key cell types [24,25,26].

DNA methylation is a mechanism during which a methyl group is attached to the cytosine base of a DNA molecule. DNA methylation is involved in important biological processes such as gene expression regulation and affects numerous mechanisms such as cell differentiation and development [27]. Additionally, it is a mechanism that may be altered by environmental factors [28]. During embryonic development, de novo DNA methylation occurs, conferred by de novo DNA methyltransferase (DNMT) [29]. Studies in mouse models have revealed that this de novo DNA methylation is critical for development as DNMT knockout embryos die in mid-gestation or shortly after birth [30]. Trophoblast cells seem to have a unique genome-wide DNA methylation pattern, characterized by large partially methylated domains (PMDs), in contrast to the highly methylated genomes of somatic cells. This feature is conserved among placental mammals and is maintained throughout pregnancy [31]. Even though the importance of imprinted DNA methylation in placentation is well-known, molecular interactions and changes in the transcriptomic profile under cytotoxic effects have not yet been established [32].

The NANOG protein is a transcription factor, and it is detected in pluripotent embryonic stem cells [33]. NANOG is essential for maintaining the self-renewal and pluripotency of human embryonic stem cells [34,35]. During the implantation of blastocysts, the corresponding mRNA is detected in the epiblast of the embryo, then its expression is restricted to primordial germ cells, and finally becomes undetectable in adult tissues [36,37]. NANOG upregulates DNMT1 through direct binding to its promoter, thereby leading to the repressed expression of p16 and p21 and consequently to genes related with development and lineage differentiation [38].

The Ishikawa cell line is a well-differentiated human endometrial adenocarcinoma cell line. Because it is characterized by the presence of estrogen and progesterone receptors, it has been used in many basic research projects in reproductive biology [39]. The aim of this study is to examine the exposure of normal and tumor-derived cells to BPS, as it seems to be of major environmental interest, and moreover to study a possible association of cell viability with the methylation-related status of the trophoblastic cells. Additionally, this study attempts to reveal the molecular interaction of NANOG with DNMT1 in trophoblastic cells as a secondary outcome. Although this interaction is already known, it has not yet been shown and proven in trophoblastic cells.

Ultimately, the present study integrates trophoblastic cells as an in vitro model to provide evidence and investigate the molecular interactions regarding the placenta-related pregnancy complications after cytotoxic exposure to BPS.

## 2. Materials and Methods

### 2.1. Study Approval and Patient Consent

The study was conducted in accordance with the Declaration of Helsinki and approved by the Institutional Ethics Committee of “Alexandra” Maternity Hospital, Medical School of the National and Kapodistrian University of Athens, with protocol identifier 7/27-07-2022. The sample collection process and the subsequent analysis was performed in the First Department of Obstetrics and Gynecology, “Alexandra” Maternity Hospital, Medical School of the National and Kapodistrian University of Athens. Informed consent for participation in the study and publication of the results was obtained from all patients included.

### 2.2. Ishikawa Cell Line Culture

Human endometrial epithelial adenocarcinoma Ishikawa cells (cell line kindly provided by the Department of Biochemistry, Medical School of Athens, Athens Greece) were cultured in DMEM (Dulbecco’s Modified Eagle Medium), supplemented with 2 mM L-glutamine, 1% non-essential amino acids, and 10% FBS (all from Invitrogen) at 37 °C and 5% CO_2_. The media of the cells were replaced with fresh medium every two or three days and subculturing was realized by using 0.05% trypsin.

### 2.3. Isolation and Expansion of Chorionic Villi

Samples (three to five milliliters) were obtained by biopsy from women who needed prenatal diagnosis, and no abnormalities were revealed by genetic analysis. All samples were centrifuged at 1800 rpm for 20 min, and after removing the supernatant, the cell pellet was washed in DMEM medium (Sigma-Aldrich Ltd., St. Louis, MO, USA) to remove blood and cell debris. After centrifugation, the cell pellet was resuspended in 5 mL of growth medium AmnioMAX-C100 (Gibco, Life Technologies, Grand Island, NY, USA), 100 U/mL penicillin, and 100  μg/mL streptomycin (Gibco, Grand Island, NY, USA), and plated in a 25 cm^2^ culture flask. Samples were incubated for 10–15 days at 37 °C in 5% CO_2_. During this period, the first colonies appeared (first stage) and the growing medium was changed every 3 days. Cells were subcultured at approximately 80% confluence by using 0.05% trypsin-EDTA (Gibco, Life Technologies, Grand Island, NY, USA). A population of fibroblast-like cells was obtained after two rounds of subculture and was observed under a microscope. The isolated cells derived from chorionic villi cultures were observed to have a fibroblast-like morphology, consistent with extravillous trophoblasts after an in vitro expansion. Although immunostaining for markers such as cytokeratin 7, hCG-beta, and vimentin was not applied, the morphological characteristics and origin of the cells support their trophoblastic lineage.

### 2.4. Chemicals

Cells obtained from the cultures were divided into plates and incubated for 24 h with different concentrations of bisphenol S (BPS). Bisphenol S (BPS) was purchased from Sigma-Aldrich Chemie Gmbh (Shanghai, China), while BPS concentrations were prepared with serial dilutions from a stock solution of 1000 mg/L BPS (powder). For both cell cultures control samples without BPS were used. BPS concentrations were added separately to cultures and their effect upon cells was observed and recorded using a microscope (Nikon eclipse te2000-5, Tokyo, Japan). In Ishikawa cells, BPS was added in concentrations of 100 pg/mL and 1000 pg/mL. In trophoblastic cells, the concentrations used ranged from 2 μg/mL to 0.1 μg/mL BPS.

### 2.5. Assessing Cell Viability

Cell viability was measured using the Countess Automated Cell Counter (Thermo Fisher Scientific, Waltham, MA, USA), with Countess cell counting chamber slides, which include Trypan Blue. The viability of the Ishikawa cells was assessed after 48 h and for trophoblasts after 24 h.

### 2.6. siRNA Transfection

Small interfering RNAs (siRNAs) targeting NANOG gene were from Origene (OriGene Technologies, Rockville, MD, USA). Trophoblastic cells were transfected with NANOG siRNA. The siTran 2. 0 Transfection Buffer (5x) was used at a final concentration of 1X, while cell cultures should be at 50% of confluence to be proceeded. After refreshing the cell culture by adding medium, a 5 μM siRNA dilution was applied. The cultures were then incubated in 37 °C and 5% CO_2_ for 18–24 h to ensure that the gene was silenced. The effect of siRNA on *NANOG* expression was evaluated using qRT-PCR.

### 2.7. Gene Expression

RNA isolation was performed using the MonarchTotal RNA MiniprepKit (New England BioLabs, Ipswich, MA, USA) following the manufacturer’s instructions. Extracted RNA samples were stored at −80 °C. For cDNA synthesis, the LunaScript RT SuperMix Kit was applied (New England BioLabs Inc.). The resulting cDNAs were stored at −20 °C. Quantification of DNMT and NANOG was performed by qPCR using the Luna Universal qPCR Master Mix (NEB). The G6PD gene was used as an internal control [40]. Table 1 presents the primer sequences for RT-PCR.

All reactions were realized on a Light Cycler 480II (Roche Applied Science, Penzberg, Germany). The 2^−ΔΔCT^ method was used to calculate the relative mRNA expression levels of DNMT gene.

### 2.8. Statistical Analysis

Frequencies and percentages were used to describe categorical data collected within the study context, while the mean was used to describe cell concentration. Differences depending on BPS, regarding trophoblasts as well as Ishikawa cells, were assessed using the Friedman’s test, followed by non-parametric multiple comparisons based on the Dunn’s criterion for type I error adjustment. Spearman’s correlation coefficient was used to assess correlations between trophoblasts as well as Ishikawa cells, with or without BPS. The choice of statistical tests was strongly based on the restriction imposed by the small sample size. The analysis was conducted using SPSS v29.0 and statistical significance was set at 0.05 in all cases.

In this study, ten different trophoblastic samples were used for cell culture and for different BPS concentrations, while the Ishikawa cell line was also cultured in ten different flasks for BPS exposure and DNMT expression. Additionally, six different trophoblastic samples were used for siRNA experiment.

## 3. Results

### 3.1. Cell Viability

**Figure 1:**
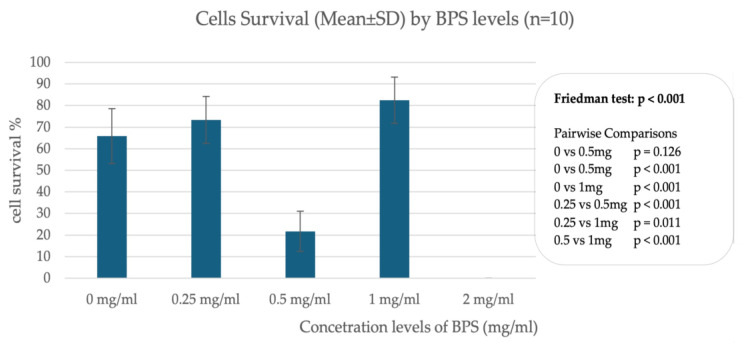
Percentage of live cells in association with different concentrations of BPS in trophoblastic cells.

In trophoblast cells, data from incubation of cells with different concentrations of BPS show differences in cell survival, indicating a nonlinear relationship between BPS concentration and percentage of live cells. A significant effect appears to occur when incubating cells with 2.0 μg/mL BPS, where trophoblast cells do not survive after 24 h. In contrast, incubation with lower concentrations such as 1.0 μg/mL and 0.25 μg/mL does not appear to affect cell viability compared to the control sample, which was incubated with deionized H_2_O. Figure 1 illustrates the percentage of live cells in association with different concentrations of BPS.

**Figure 2:**
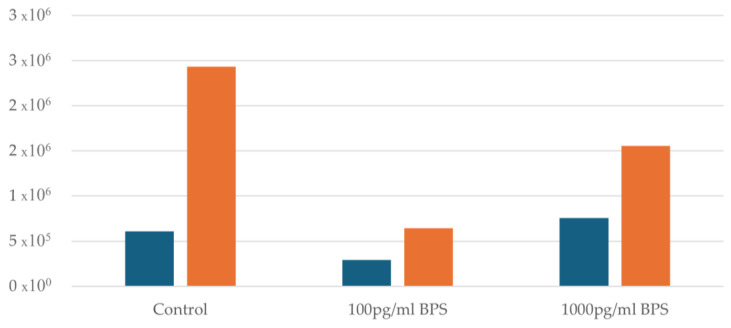
Cell concentration before and after the addition of BPS. Blue columns indicate cell concentration before the addition of BPS, whereas yellow columns after the addition of BPS.

Regarding the Ishikawa cell lines, the effect of the two different concentrations (100 pg/mL and 1000 pg/mL) of bisphenol S (BPS) being added was examined and compared with control cultures (without addition of BPS) of Ishikawa cells to test whether BPS may prove to be toxic to this specific cell line. An initial cell viability measurement was performed before adding BPS to all samples. Afterwards, samples were divided into control flasks of 100 pg/mL and 1000 pg/mL BPS flasks. In all samples after the addition of BPS and after 48 h, a distinct increase in cell concentration is observed. Figure 2 illustrates the cell concentration before and after the addition of BPS.

### 3.2. DNMT1 Gene Expression

**Figure 3:**
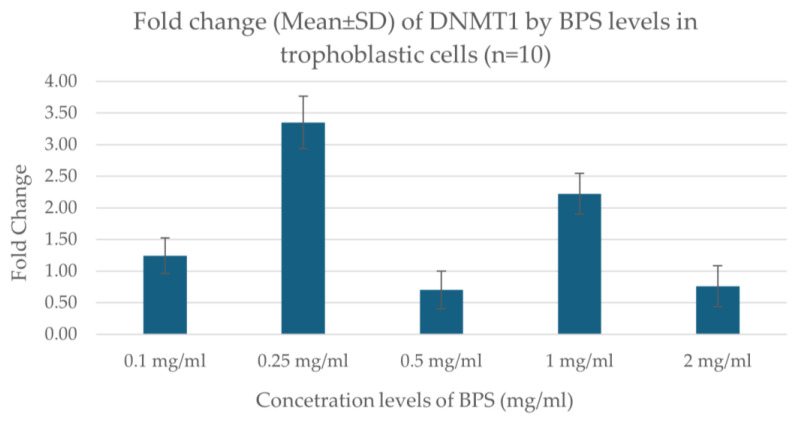
Fold change in *DNMT1* expression in association with different concentration levels of BPS in trophoblastic cells.

Real-time PCR results showed that the expression of the *DNMT1* gene varies depending on the concentration of BPS in trophoblastic cells. Specifically, the expression showed a higher increase at the concentration of 0.25 μg/mL BPS, indicating a possible stimulation of gene expression at this dose. At the lowest concentrations (0.1 μg/mL), a smaller increase was observed, while at the highest concentrations (0.5, 1.0, 2.0 μg/mL), a decrease in expression was noted. The data indicate a nonlinear effect of BPS on gene expression, possibly related to toxicity or cellular response at the different concentrations. Figure 3 illustrates the fold change in DNMT1 expression regarding the concentration levels of BPS in trophoblastic cells.

**Figure 4:**
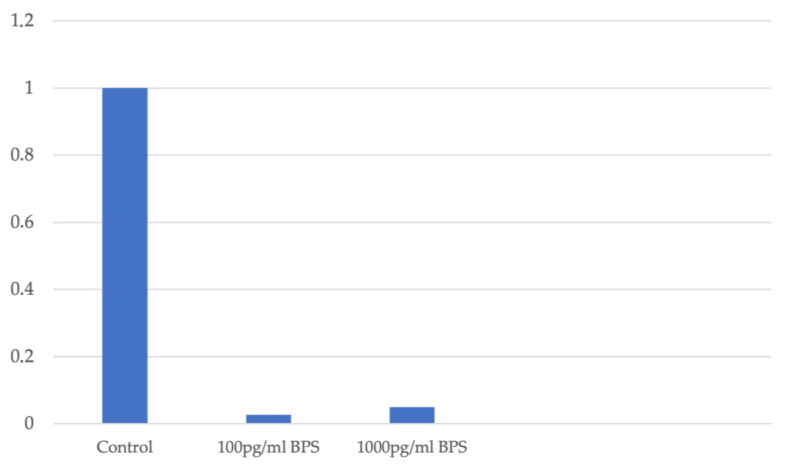
Fold change in DNMT1 expression in association with different concentration levels of BPS in Ishikawa cell lines.

In the Ishikawa cell line, the fold change measurement of DNMT1 expression is decreased independently of the cell viability, which is not affected by addition of BPS. A similar decrease in DNMT1 expression is observed after the addition of BPS. Figure 4 illustrates the fold change in *DNMT1* gene expression in association with the different concentration levels of BPS used in Ishikawa cell lines.

**Figure 5:**
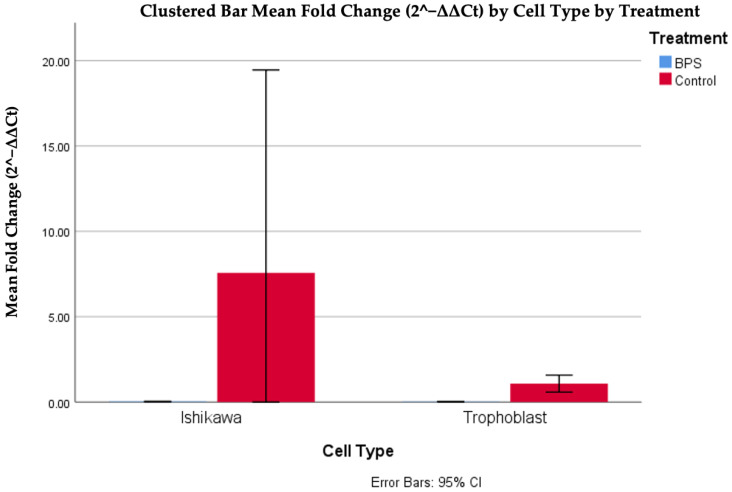
Cp DNMT1 expression in different cells, with or without the addition of BPS.

The analysis of the data indicated a statistically significant difference between fold changes in trophoblasts with and without BPS, in a nonlinear manner, as high values were observed in the case of BPS presence (*p* < 0.001). The difference based on the presence of BPS was also observed between Ishikawa cells, where higher values were also observed in the absence of BPS. Finally, it should also be noted that a higher expression of the DNMT1 gene was observed in Ishikawa cells compared to trophoblastic cells in the absence of BPS. Figure 5 and Table 2 illustrate the differences in DNMT1 expression in different cells, with or without the addition of BPS.

**Figure 6:**
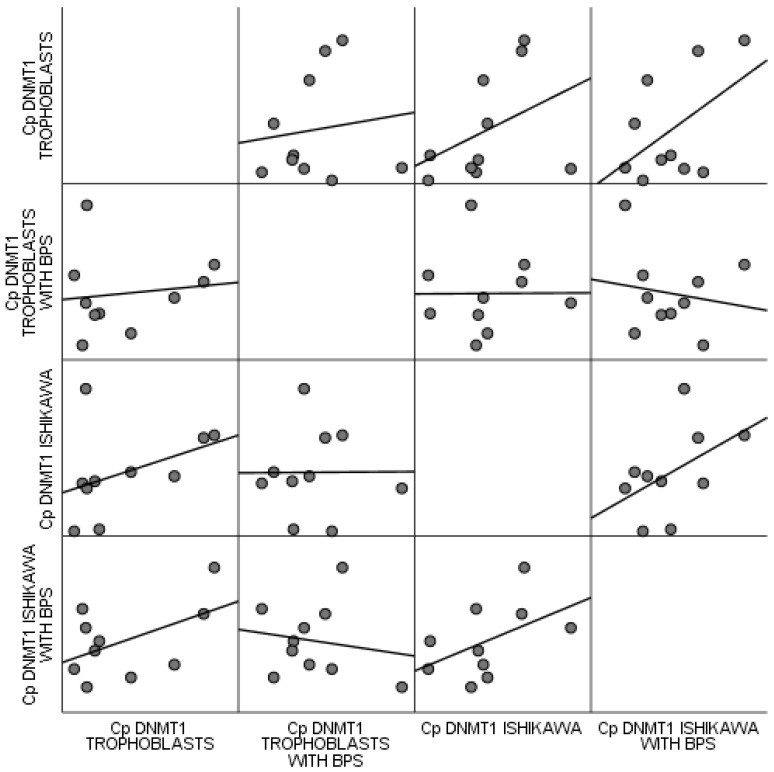
Scatter matrix of correlations observed between trophoblastic cells and the Ishikawa cell line regardless of BPS.

No statistically significant correlations were observed between trophoblastic cells and the Ishikawa cell line, regardless of BPS. All estimated *p* values were above 0.1, based on Spearman’s correlation coefficient. These correlations are attributed by the scatter matrix of Figure 6.

### 3.3. NANOG Silencing in Trophoblastic Cells

The detection of *NANOG* in trophoblastic cells was verified using real-time PCR, indicating a low but stable expression of the gene, while a housekeeping gene (G6PD) was also detected. After applying the siRNA technique, the *NANOG* expression was not detected, while the *G6PD* gene was still present. Silencing the *NANOG* gene resulted in a reduced expression of *DNMT1*, while *G6PD* gene was still detected.

## 4. Discussion

This study investigates the cytotoxic and molecular effects of bisphenol S (BPS) on trophoblastic cells and the Ishikawa cell line, with a focus on cell viability and the regulation of DNMT1 and NANOG expression. The findings provide a novel insight into the differential cellular responses to BPS and underscore the potential implications for early pregnancy and placental development.

BPA and BPS are the most abundant bisphenol substances detected in humans. There is growing evidence that BPS exposure can affect human health [41,42]. Moreover, previous studies show that BPS can increase estrogen-responsive gene expression in the ovaries and uterus [43]. The placenta is known to be involved in the development of the fetus and to play multiple roles in nutrient, gas, and waste exchange and in the production of hormones and cytokine factors. Even though the placenta detoxifies certain chemicals, it can also be affected by EDCs like BPS. Exposure to BPS is primarily dietary and can be transmitted from mother to offspring via the placenta. Consequently, it is considered as a major environmental risk during pregnancy.

Previous animal studies in rodents have focused either on the transcriptomic responses or effects on placental morphology after BPA exposure [44,45]. However, there are fewer reports on the effects of BPS. Our data confirmed a dose-dependent cytotoxic effect of BPS on trophoblastic cells, with significant results observed at concentrations ≥2.0 μg/mL. In contrast, lower concentrations (≤1.0 μg/mL) did not significantly impair viability. This aligns with previous reports that highlight the sensitivity of placental cells to EDCs like BPS, which may compromise trophoblast function and pregnancy outcomes [8,9,10]. The nonlinear response observed may be related to a biphasic mechanism of BPS action, consistent with its endocrine-disrupting properties; low doses may induce subtle molecular changes while higher doses are related to cytotoxicity.

Concerning the effect of BPS on the Ishikawa cell line, a study by Kimberly et al. reports that BPS increased the total number of viable cells and stimulated migration in endometrial epithelial cells, specifically in the Ishikawa cell line. The proliferative effects of BPS were also observed in mouse uteri and in the endometrial glandular epithelium—the primary site of endometrial hyperplasia [46]. Our results in the Ishikawa cell line are in accordance with the previous findings, as they displayed increased cell proliferation upon exposure to BPS, regardless of concentration. This proliferative response may be related to the presence of estrogen receptors in Ishikawa cells, which trigger a mitogenic signaling in the presence of estrogenic compounds such as BPS [47]. As the response between normal trophoblasts and neoplastic endometrial cells is differentiated, a context-specific action of BPS, related to the cellular morphology and microenvironment, can be proposed.

DNA methylation is one of the epigenetic mechanisms that plays a critical role in preimplantation embryo development and is mainly characterized by DNA methyltransferase (DNMT) enzymes. DNMT1 is the methyltransferase that ensures the activation and repression of developmental related genes. It has been reported that the lack of DNMT enzymes results in infertility, epigenetic abnormalities, or embryonic demise [48]. Our study shows that BPS exposure significantly influences *DNMT1* gene expression in trophoblastic cells. More specifically, *DNMT1* expression peaked at 0.25 μg/mL BPS, followed by a sharp decline at higher concentrations, suggesting that moderate levels of BPS may initially induce *DNMT1* expression, a possible compensatory epigenetic response. On the other hand, elevated levels of BPS may inhibit gene expression through cytotoxic mechanisms, inducing, through epigenetic mechanisms, cellular dysregulation, and contributing to placental dysfunction, through altered trophoblast differentiation.

Although there are a few studies related to *DNMT1* expression and Ishikawa cells, there are no previous reports about *DNMT1* expression before and after the use of BPS [49,50]. Based on our results, we could hypothesize that BPS enters Ishikawa cells through passive diffusion and by mimicking estrogen, binds to estrogen receptors (ERα/ERβ), and suppresses *DNMT1* gene expression. Downregulating *DNMT1* mRNA leads to passive demethylation during DNA replication and consequently to altered gene expression.

In this study, silencing of the *NANOG* gene in trophoblastic cells using siRNA confirmed the presence of a regulatory interaction between *NANOG* and *DNMT1*. Real-time PCR results revealed a low but stable expression of the *NANOG* gene in trophoblasts, consistent with previous reports [33,34,35,36,37]. After successful silencing of *NANOG*, *DNMT1* expression was also reduced, while the *G6PD* gene was detectable, confirming the efficiency of the knockdown procedure. These findings are in accordance with prior reports on mesenchymal and embryonic stem cells which reveal that NANOG directly upregulates *DNMT1* by binding to its promoter region [38]. The observed downregulation of *DNMT1* after *NANOG* silencing suggests that a similar epigenetic regulatory axis is present in human trophoblastic cells. According to previous reports, the *NANOG–DNMT1* axis may represent a key mechanism by which trophoblastic epigenetic integrity is preserved [29,30,31,32]. Disruption of this axis—by gene silencing or environmental action such as BPS exposure—may alter the differentiation, function, or invasiveness of trophoblasts, essential mechanisms for placental development and successful pregnancy outcomes.

Strengths of the present study constitute the utilization of trophoblastic cells and cell lines in the investigation of the cytotoxic effects of BPS. Furthermore, this study, apart from the viability assessment, delves into the differential cellular responses to BPS in the scope of early pregnancy. Moreover, this is, to our knowledge, the first study that reports *NANOG* silencing in trophoblastic cells and provides data on the effect of the silencing and the future implications in an ongoing pregnancy.

However, while cell lines are valuable research tools, their altered biology, the probability of contamination, and inability to fully replicate in vivo conditions constitute limitations of their use. Hence, the extrapolation of in vitro results to humans should be undertaken with caution. Furthermore, the use of phenol red-containing media may have introduced estrogenic interference, potentially confounding the observed cellular responses to bisphenol S (BPS). Future experiments using phenol red-free media are recommended to validate these findings. Furthermore, while this study focused on mRNA expression profiles of key genes such as *DNMT1* and *NANOG*, the translation of these changes into functional protein alterations and their downstream biological effects remain to be explored. Subsequent investigations should include protein-level validation and pathway interaction analyses to elucidate the mechanistic implications of the observed gene expression changes. Additionally, although DNA methylation is central to the study’s hypothesis, no direct methylation analysis was performed. Future studies should incorporate gene-specific or genome-wide methylation assays to verify the functional relevance of transcriptional changes. Another limitation is the absence of a time-course design, which would be critical in characterizing the dynamic cellular response to BPS exposure and this is proposed for future studies. Additionally, the inclusion of known DNA methyltransferase modulators, such as 5-azacytidine, as positive controls, could strengthen the mechanistic insights and confirm the specificity of the observed epigenetic effects. Lastly, the impact of environmental exposures and mainly the action of EDCs may offer insight into the cellular and molecular origins of pregnancy complications such as fetal growth restriction and miscarriage.

## 5. Conclusions

In conclusion, the results of this study highlight the cytotoxic effects of BPS and consequently its action on trophoblast viability. The results of NANOG-DNMT1 gene expression related to BPS exposure reinforces our understanding of EDC-induced placental dysfunction. Further investigation into the developmental and reproductive toxicity of BPS in relation to placental development should provide new insights on the related cellular and molecular mechanisms.

## Figures and Tables

**Table 1:** Primer sequences for RT-PCR.

Gene	Sequence	
DNMT	Forward	5′-AGGTGGAGAGTTATGACGAGGC-3′
	Reverse	5′-GGTAGAATGCCTGATGGTCTGC-3′
NANOG	Forward	5′ AGA-TGC-CTC-ACA-CGG-AGA-CTG 3′
	Reverse	5′ CAT-CTG-CTG-GAG-GCT-GAG-GTA 3′
G6PD	Forward	5′-TGGACCTGACCTACGGCAACAGATA-3′
	Reverse	5′-GCCCTCATACTGGAAACCC-3′

**Table 2:** Descriptive characteristics of the different cells depending on the addition of BPS.

Cell Type	Treatment	ΔCt	Mean ΔCt (Control)	ΔΔCt	Fold Change (2^−ΔΔCt^)
Trophoblast	Control	−5.05	−5.008	−0.042	1.029540083
Trophoblast	Control	−5.19	−5.008	−0.182	1.134455485
Trophoblast	Control	−5.32	−5.008	−0.312	1.241427492
Trophoblast	Control	−5.63	−5.008	−0.622	1.53900722
Trophoblast	Control	−3.85	−5.008	1.158	0.44813335
Trophoblast	BPS	0.03	−5.008	5.038	0.030437633
Trophoblast	BPS	−0.08	−5.008	4.928	0.032849153
Trophoblast	BPS	0.53	−5.008	5.538	0.021522657
Trophoblast	BPS	0.27	−5.008	5.278	0.025772923
Trophoblast	BPS	0.3	−5.008	5.308	0.025242524
Ishikawa	Control	−9.84	−5.544	−4.296	19.64377094
Ishikawa	Control	2.57	−5.544	8.114	0.003609463
Ishikawa	Control	−5.78	−5.544	−0.236	1.17772279
Ishikawa	Control	−9.57	−5.544	−4.026	16.2909632
Ishikawa	Control	−5.1	−5.544	0.444	0.735093668
Ishikawa	BPS	−0.4	−5.544	5.144	0.028281452
Ishikawa	BPS	−0.18	−5.544	5.364	0.024281477
Ishikawa	BPS	−0.6	−5.544	4.944	0.032486857
Ishikawa	BPS	−0.32	−5.544	5.224	0.026755884
Ishikawa	BPS	−1.01	−5.544	4.534	0.043164827

## Data Availability

The raw data supporting the conclusions of this article will be made available by the corresponding author on request.
